# DNA-MVA-protein vaccination of rhesus macaques induces HIV-specific immunity in mucosal-associated lymph nodes and functional antibodies

**DOI:** 10.1016/j.vaccine.2016.12.060

**Published:** 2017-02-07

**Authors:** Gerald K. Chege, Wendy A. Burgers, Tracey L. Müller, Clive M. Gray, Enid G. Shephard, Susan W. Barnett, Guido Ferrari, David Montefiori, Carolyn Williamson, Anna-Lise Williamson

**Affiliations:** a Division of Medical Virology, Department of Pathology, Faculty of Health Sciences, University of Cape Town, Cape Town, South Africa; b Division of Immunology, Department of Pathology, Faculty of Health Sciences, University of Cape Town, Cape Town, South Africa; c Department of Medicine, Faculty of Health Sciences, University of Cape Town, Cape Town, South Africa; d Institute of Infectious Disease and Molecular Medicine, Faculty of Health Sciences, University of Cape Town, Cape Town, South Africa; e South African Medical Research Council, Cape Town, South Africa; f Glaxo Smith Kline, Cambridge, MA, USA; g Duke University Medical Center, Durham, NC, USA; h National Health Laboratory Service, Groote Schuur Hospital, Cape Town, South Africa

**Keywords:** Poxvirus/protein, HIV vaccines, Nonhuman primates

## Abstract

Successful future HIV vaccines are expected to generate an effective cellular and humoral response against the virus in both the peripheral blood and mucosal compartments. We previously reported the development of DNA-C and MVA-C vaccines based on HIV-1 subtype C and demonstrated their immunogenicity when given in a DNA prime-MVA boost combination in a nonhuman primate model. In the current study, rhesus macaques previously vaccinated with a DNA-C and MVA-C vaccine regimen were re-vaccinated 3.5 years later with MVA-C followed by a protein vaccine based on HIV-1 subtype C envelope formulated with MF59 adjuvant (gp140Env/MF59), and finally a concurrent boost with both vaccines. A single MVA-C re-vaccination elicited T cell responses in all animals similar to previous peak responses, with 4/7 demonstrating responses >1000 SFU/10^6^ PBMC. In contrast to an Env/MF59-only vaccine, concurrent boosting with MVA-C and Env/MF59 induced HIV-specific cellular responses in multiple mucosal associated lymph nodes in 6/7 animals, with high magnitude responses in some animals. Both vaccine regimens induced high titer Env-specific antibodies with ADCC activity, as well as neutralization of Tier 1 viruses and modest Tier 2 neutralization. These data demonstrate the feasibility of inducing HIV-specific immunity in the blood and mucosal sites of viral entry by means of DNA and poxvirus-vectored vaccines, in combination with a HIV envelope-based protein vaccine.

## 1 Introduction

It is widely accepted that a successful HIV vaccine will need to elicit both effective T cell responses and functional antibodies [1], [2]. Since the lymphoid tissues in the gut mucosa and other mucosa-associated lymphoid tissues (MALT) are the primary sites of HIV amplification [3], [4], [5], [6], potent anti-HIV cellular responses such as CD8+ T cell-mediated and antibody-dependent cellular cytotoxic (ADCC) responses would be required at these sites to target and destroy virus-infected cells in order to stop the virus disseminating throughout the host. While the majority of T cell-based vaccines, even while eliciting robust T cell responses, may fail to prevent the infection from establishing in exposed individuals, but rather control viral replication to sufficiently delay the onset of AIDS [2], [7], [8], a recent study showed that some T cell vaccines may potentially provide protection or, if sufficiently potent, enable immune clearance over time [8]. Generation of effective and long-lived T cell responses in the peripheral blood and mucosal compartments therefore remains a key goal in HIV vaccine strategies.

In addition to cellular immunity, generation of high titers of HIV Env-specific antibodies, including neutralizing (Nab) and non-neutralizing (nonNab), is a crucial goal for developing effective HIV vaccines [2], [9], [10]. The induction of Nab, particularly broadly neutralizing antibodies (bNab) that have the capacity to cross-neutralize 50–90% of transmitted viruses would be desirable for a candidate HIV vaccine, but such antibodies are challenging to generate by vaccination. A more realistic goal may be the generation of nonNab, whose importance was underscored in the RV144 trial that demonstrated modest protection from HIV infection [11]. Protection was associated with vaccine-generated IgG antibodies to the V1/V2 region of gp120 [2], [10] in the absence of bNab, with only weak tier 1 Nab being detected [12]. Initial case-control analyses showed that IgG antibodies to V1/V2 inversely correlated with risk of HIV-1 infection but in vaccinees with high levels of Env-specific IgA, IgG avidity, antibody-dependent cellular cytotoxicity (ADCC), Nab, and Env-specific CD4+ T cells, there was no correlation between these variables and infection [13]. However, secondary immune correlates analysis demonstrated that, in the setting of low IgA antibodies, high levels of ADCC-mediating IgG antibodies correlated inversely with risk of HIV infection [14], and in particular those belonging to IgG1 and IgG3 subclasses [14], [15], suggesting that the observed protection in RV144 was, at least partially, due to ADCC-mediating antibodies [16]. A number of nonhuman primate (NHP) studies have also reported protection against SIV acquisition in the absence of bNab [17], [18], [19], [20], providing further evidence that functional vaccine-induced Env-specific antibodies may indeed provide protection against HIV acquisition even in the absence of bNab. Furthermore, nonNab of different specificities can act in concert for increased antiviral activity, through recognition of virus-infected cells, infectious virion capture and ADCC [21]. Intense efforts to generate bNab via vaccination continue, through innovative immunogen design and immunization regimens [22], as well as seeking to harness CD4+ T cell responses to support long-lasting antibody-producing plasma and memory B cells, via follicular helper T cells (TFH), that appear to be crucial for induction of Nab [23], [24].

The RV144 trial demonstrated partial protection from HIV infection using a poxvirus-protein immunization regimen based on subtypes B and CRF01_AE [11]. HIV-1 subtype C (HIV-1C) is responsible for 50% of infections globally [25], and we have previously described the development of a candidate vaccine regimen comprising of two multigene recombinant vaccines, a DNA vaccine (DNA-C) and an MVA vaccine (MVA-C), expressing matched HIV-1C proteins (Gag, RT, Tat, Nef, Env) [26], [27], [28]. The candidate vaccines, given as a DNA-C/MVA-C prime/boost combination to elicit T cell responses, were developed for evaluation in a phase 1 clinical trial (HVTN 073/ SAAVI 102) [29], that was later modified to include a protein envelope vaccine boost (HVTN 073E/SAAVI 102E) following the finding of partial protection in the RV144 efficacy trial [11]. In the current study, we evaluated the SAAVI vaccine regimen in combination with an HIV-1C gp140ΔV2 Env protein in nonhuman primates (NHP). The Env protein was previously shown to induce higher titer neutralizing antibodies in rabbits compared to intact gp140 [30]. This study in rhesus macaques, designed based on the concept of RV144, was intended to complement the clinical trial (HVTN 073E/SAAVI 102E) [29] and to further investigate the induction of vaccine-specific cellular responses in mucosal compartments, and functional antibodies in the peripheral blood. It also examined the potential of MVA-based vaccines as a booster after a long interval since the priming vaccination.

## 2 Materials and methods

### 2.1 DNA-C and MVA-C vaccines

The construction and immunogenicity of the candidate HIV vaccines, SAAVI DNA-C and SAAVI MVA-C, have previously been described in detail [26], [27], [28]. Briefly, the two candidate vaccines contained identical inserts based on HIV-1 subtype C viruses from South Africa (Du422 and Du151), expressing five HIV proteins (Gag, RT, Tat, Nef and Env).

### 2.2 Env protein vaccine (gp140Env/MF59)

The HIV-1 gp140 ΔV2 Env protein vaccine was produced by Novartis Vaccines & Diagnostics Inc (now GSK, Cambridge, MA) and comprised a recombinant oligomeric V2-deleted envelope (gp140) protein derived from an HIV-1C South African primary isolate (TV1) [30] and produced in Chinese Hamster Ovary cells. The protein was formulated with MF59 adjuvant [31] (Novartis Vaccines & Diagnostics Inc, MA) just prior to administration. MF59 adjuvant is an oil-in-water emulsion with a squalene internal oil phase and a citrate buffer external aqueous phase. MF59 adjuvant has recently been shown to promote germinal center B cell differentiation and persistence in response to vaccination [30], [31].

### 2.3 Macaque immunizations

**Fig. 1:**
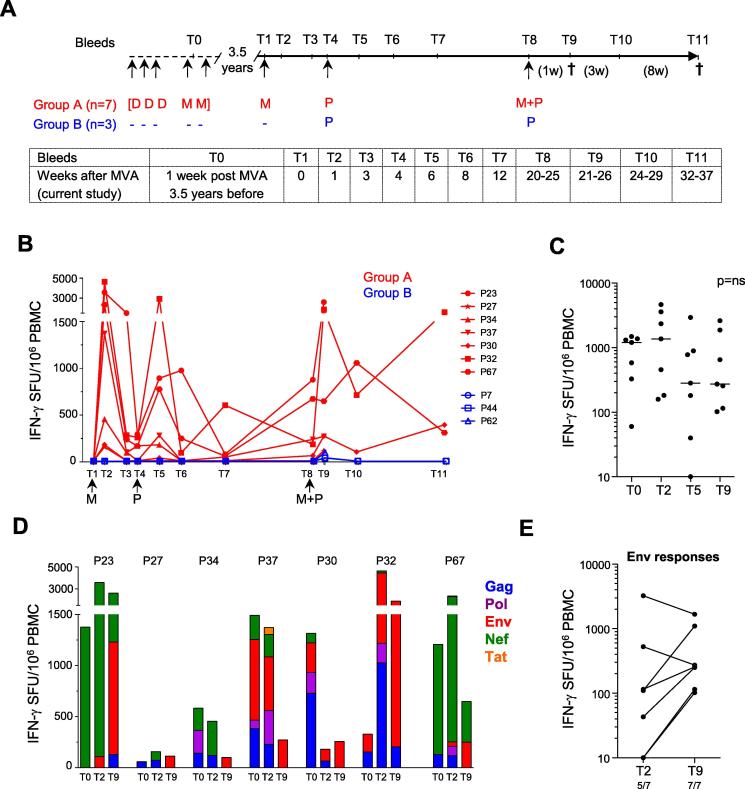
Dynamics, magnitude and breadth of HIV-specific T cells induced by MVA-C and gp140Env/MF59 vaccination in the blood of rhesus macaques. (A) Vaccination schedule for SAAVI DNA-C (DNA-C), SAAVI MVA-C (MVA-C) and gp140Env/MF59 vaccines. Seven animals (Group A) were primed with three doses of DNA-C (4 mg each; intramuscularly), 4 weeks apart, followed 2 months later by two doses of MVA-C (10^9^ pfu; intramuscularly), 4 weeks apart (DDDMM). 3.5 years later, these animals were boosted with MVA-C (M; 10^9^ pfu), followed 4 weeks later by TV1 gp140ΔV2 Env protein (100 mg) formulated in MF59 adjuvant (P; gp140Env/MF59; intramuscularly), then boosted further a median of 18 (range 16–21) weeks later with gp140Env/MF59 in combination with MVA-C (M + P). A control group of 3 animals (Group B) that had not received the initial vaccination regimen was vaccinated intramuscularly twice with the gp140 Env/MF59 alone. The second gp140Env/MF59 was given at a median of 18 (range 16–21) weeks after the first protein vaccination corresponding with the gp140En/MF59 and MVA-C + gp140Env/MF59 vaccinations in Group A animals. Serum and peripheral blood mononuclear cells (PBMC) were obtained at various time points (T0 to T11). Animals were euthanized (†) at 1 or 12 weeks after the last vaccination for harvesting of mucosal samples and isolation of mononuclear cells. (B) Dynamics of vaccine-induced IFN-γ ELISPOT responses. Arrows indicate timing of vaccinations with MVA-C (M), gp140Env/MF59 (P) and either gp140Env/MF59 alone (Group B) or both MVA-C and gp140Env/MF59 (Group A) (M + P). Responses are expressed as net (background-subtracted) spot forming units (SFU) per 10^6^ PBMC performed on fresh cells, and are the sum of Gag, Pol, Env, Nef and Tat peptide pool responses per animal. (C) Magnitude of vaccine-induced IFN-γ ELISPOT responses (Group A). Peak responses after vaccination are shown, which occurred at T0 (1 week after the first MVA-C [with the exception of P32, whose peak response occurred at week 1 week after the second MVA-C]), T1 (1 week after the booster MVA-C that was given 3.5 years after the last MVA-C during the initial DNA-C/MVA-C), T5 (2 weeks after the first gp140Env/MF59) or T9 (1 week after MVA-C + gp140Env/MF59). The horizontal lines indicate the median responses, of which no significant (ns) differences were observed. (D) Breadth of vaccine-induced IFN-γ ELISPOT responses (Group A). The breadth of responses are represented by the relative proportions of the magnitudes of responses to Gag, Pol, Env, Nef and Tat at T0, T2 and T9. (E) Magnitude of Env-specific IFN-γ ELISPOT responses. Magnitudes of responses specific for Env vaccine immunogen, at the peak time point after MVA-C (T2) and second gp140Env/MF59 which was given together with MVA-C (T9) are shown for animals in Group A. The frequency of responders is indicated below the graph. A cut-off value of 40 SFU for positive responses was used. Medians are shown as a horizontal line. Statistical comparisons were determined using a non-parametric Mann-Whitney test (ns = not significant at P < 0.05).

Ten rhesus macaques (*Macaca mulatta*) of Chinese origin were used for this study. The animals were housed at the South African Medical Research Council’s (MRC) animal facility in Cape Town. They were maintained in accordance with the South African national guidelines for Use of Animals for Scientific Purposes (SANS Code 10386) and the EU Directive 2010/63/EU for animal experiments. Seven animals that had been previously vaccinated by priming three times with DNA-C (4 mg) and boosting twice with MVA-C (10^9^ pfu) 3.5 years prior were boosted in this study with MVA-C (10^9^ pfu), followed by gp140Env/MF59 (100 mg protein formulated in MF59 adjuvant). Finally, a concurrent boost with both vaccines was given, as detailed in Fig.1A (Group A). The vaccines were administered separately via the intramuscular route into alternative quadriceps muscles. Three additional animals that had not received the initial vaccination regimen were immunized twice with the gp140Env/MF59 alone (Fig.1A, Group B). Blood samples were obtained at various time points and spleen and various mucosal-associated lymph nodes (bronchial, iliac, inguinal, and mesenteric) were harvested at the experimental endpoint. The experimental protocol was reviewed and approved by the Animal Ethics Committee of the University of Cape Town (Protocol Refs: 006/032 and 008/014).

### 2.4 Isolation of PBMC, splenocytes and LNMC

Peripheral blood mononuclear cells (PBMC) were isolated from heparinized whole blood by standard ficoll gradient centrifugation. To isolate splenocytes from spleens harvested at necropsy, the mesenchymal tissues were mashed through a metal sieve with 100 μm diameter pores. The resultant fine pulp was diluted in culture medium and after removal of debris, the splenocyte suspension was further disrupted by passing through a fine-bore needle (e.g. G25) and finally through a 70-μm cell strainer (BD Biosciences). Cells were washed in complete culture medium (R10; RPMI-1640 supplemented with 10% fetal bovine serum and penicillin-streptomycin – all from Gibco, Invitrogen). Red blood cells were lysed using ammonium chloride solution (Sigma-Aldrich). To extract lymph node mononuclear cells (LNMC), whole lymph nodes were carefully trimmed to remove fat and fibrous tissues and then lacerated with a scalpel blade to disrupt the capsular coating. The cells were teased out of the mesenchymal tissue into the culture medium using a scalpel blade. The cell suspension was clarified using a 70-μm cell strainer.

Freshly isolated PBMC, splenocytes and LNMC that were not used immediately in IFN-γ ELISPOT assays were resuspended in fetal bovine serum (FBS, Gibco, Invitrogen) containing 10% dimethyl sulfoxide (DMSO; Sigma-Aldrich) and cryopreserved in liquid nitrogen until use.

### 2.5 IFN-γ ELISPOT assay

Freshly isolated PBMC, splenocytes and LNMC were resuspended in R10 medium and tested for reactivity to peptide pools based on the vaccine-expressed HIV antigens, using a standard IFN-γ ELISPOT assay, as previously described [32], [33], [34]. Two hundred thousand cells per well, in triplicates, were stimulated with pools of synthetic peptides corresponding to subtype C HIV-1 Gag, Pol, Env, Nef and Tat. The peptides were 15-18mers (overlapping by 10 amino acids) and were obtained from the NIH AIDS Research Reagent Program (Bethesda, MD). Spots were analyzed using a Cellular Technology Ltd (CTL) analyzer. A cut-off value for a positive response based on pre-vaccination peptide reactivity was set at 40 SFU/10^6^ cells after subtraction of the background response. Phytohaematoglutinin-M (PHA-M, Sigma-Aldrich) was used as a positive control and a cut-off value of >500 SFU/10^6^ PBMC was used to validate the assay.

### 2.6 B-ELISPOT assay

HIV-1 Env-specific IgG-secreting cells were quantified in a B-cell ELISPOT assay using a human IgG ELISpot kit (Mabtech) according to manufacturer’s guidelines. Briefly, B cells were prepared by stimulating thawed cryopreserved PBMC with a B cell polyactivator (R848; TLR 7/8 agonist; supplied in the kit) for 5 days. A day before the cells were ready, ELISPOT plates were incubated overnight at 4 °C with Env gp140 (TV1 gp140ΔV2) in phosphate-buffered saline (PBS) at 1 μg/well (pre-titrated). Positive control wells were incubated with anti-human IgG monoclonal capture antibody while negative control wells were incubated with PBS only. After washing off unbound protein or antibody, the wells were incubated with culture medium containing 10% fetal bovine serum for 30 min. The culture medium was removed and the 5-day stimulated PBMC were added in triplicates at 200,000/well (gp140-coated and negative control wells) or 25,000/well (anti-human IgG antibody-coated wells). The plates were incubated for 24 h in a humidified incubator at 37 °C with 5% CO_2_. After washing off the cells, the plates were incubated for 2 h at room temperature with anti-IgG secondary antibody conjugated to biotin. Spots were developed as in a standard IFN-γ ELISPOT assay using streptavidin/horseradish peroxidase and BCIP/NBT substrate and analyzed using a CTL analyzer. A cut-off value for a positive response was set at 30 SFU/10^6^ PBMC after subtraction of the background response (cells in wells coated with PBS alone).

### 2.7 Antibody ELISA

HIV Env-specific IgG responses were measured in a standard ELISA. Plates were coated with gp140 (TV1 gp140ΔV2; Novartis Vaccines & Diagnostics Inc; Cambridge, MA) at 10 ng/well (pre-titrated). Serially diluted sera, starting at 1:50 were tested together with a negative control serum, consisting of a pool of pre-immune monkey sera at 1:50 dilution. Serum IgG antibodies were detected using rabbit anti-monkey IgG horseradish peroxidase (Sigma-Aldrich) diluted 1:2000 and the colour developed with tetramethyl-benzidine (KPL; Gaithersburg, MA). Data are presented as endpoint antibody titer, defined as the reciprocal of the highest serum dilution with an absorbance that was equal or greater than two times the mean absorbance of the negative control. A titer <50 was considered a negative antibody response.

### 2.8 HIV-1 neutralizing antibody assay

HIV-1 neutralizing antibodies were measured against Tier 1 and Tier 2 viruses using the TZM-bl assay as previously described [35]. MN.3, SF162.LS, MW965.26, TV1.21 and DU151.2 pseudoviruses were used, with all pseudoviruses produced in 293T cells. Serial serum dilutions starting at 1:20 were tested using a fully automated optimized TZM-bl assay system and the endpoint titers were generated automatically based on 50% reduction in RLU values compared with RLU values from the virus control wells after subtraction of background RLU (cell control wells). The endpoint titer was defined as the reciprocal of the serum dilution corresponding to the 50% inhibition and a titer <20 was considered a negative antibody response.

### 2.9 ADCC assay

ADCC activity in the sera was evaluated by measuring the Granyzme B (GzB) and luciferase activities using gp120-coated target cells as previously described [36,37]. HIV-1 subtype C TV-1 gp120-coated target cells were used and sera were tested in duplicates in 4-fold serial dilutions starting at 1:100. PBMC obtained from healthy HIV-seronegative donors served as the source of effector cells and used at an effector/target ratio of 33:1. Sera from healthy human and from HIV-1-infected donors were used as negative and positive controls respectively. GzB activity is reported as the percentage of GzB positive cells, which represents the frequency of target cells recognized by effector cells in the presence of ADCC-mediating antibodies. The background GzB activity (target and effector cells in the absence of serum) was subtracted from all samples and a cut-off for positive responses was set at 8%. ADCC antibody titers were generated from the GzB activity results and defined as the reciprocal of the last dilution at which the %GzB activity was equal to 8%. A titer <100 was considered a negative ADCC response.

### 2.10 Data analysis

Statistical analyses were performed using Prism version 5.0 (GraphPad). Statistical comparisons to assess the significance levels between median ELISPOT magnitudes were determined using a non-parametric Mann-Whitney *U* test while those between antibody titers were performed using one-way ANOVA Friedman test with Dunn’s post test for multiple comparisons. All tests were two-tailed and p-values less than 0.05 were considered significant.

## 3 Results

### 3.1 MVA-C effectively boosts vaccine responses primed 3.5 years prior

We tested the immunogenicity of a poxvirus-protein vaccine regimen in seven rhesus macaques that had previously been vaccinated with DNA-C and MVA-C (Fig.1A, Group A). To measure vaccine-induced T cell immunity, we performed an IFN-γ ELISPOT using peptide pools based on the vaccine-expressed HIV antigens. No responses were detectable at baseline, 3.5 years after animals had previously been vaccinated, but a single MVA-C boost induced high magnitude cellular responses in the majority of animals (Fig.1B). Response magnitudes were similar to previous peak responses during the initial course of vaccination (T0), reaching a median of >1000 SFU/10^6^ PBMC (Fig.1C). In addition, MVA-C re-vaccination after 3.5 years induced responses largely of the same specificity of those induced at initial vaccination (Fig.1D). The breadth of the response prior to Env protein boosting (T2) varied widely between animals, from recognition of two to all five vaccine-expressed HIV proteins, a likely reflection of the outbred nature and differential MHC expression of the animals. Nef responses were detectable in 6/7 animals, dominating the response at T2. It is noteworthy that whilst a combined MVA-C and gp140Env/MF59 vaccination did not further increase the cumulative response magnitudes (T9, Fig.1C and D) compared to the single MVA-C boost, Env-specific T cell responses were boosted in 5/7 animals, with Env T cell responses being detected in all animals one week after the gp140Env/MF59 booster at T9 (Fig.1D and E). Gag responses were detectable in all animals at either T2 or T9. A second group of animals received two vaccinations with protein vaccine only (gp140Env/MF59) without prior priming (Fig.1A, Group B). As expected, protein alone induced only Env-specific cellular responses that were of low magnitude and short-lived, and only emerged after the second protein vaccination (Fig.1B).

### 3.2 MVA-C/Env protein vaccination elicits T cell responses in multiple mucosal associated lymph nodes

**Fig. 2:**
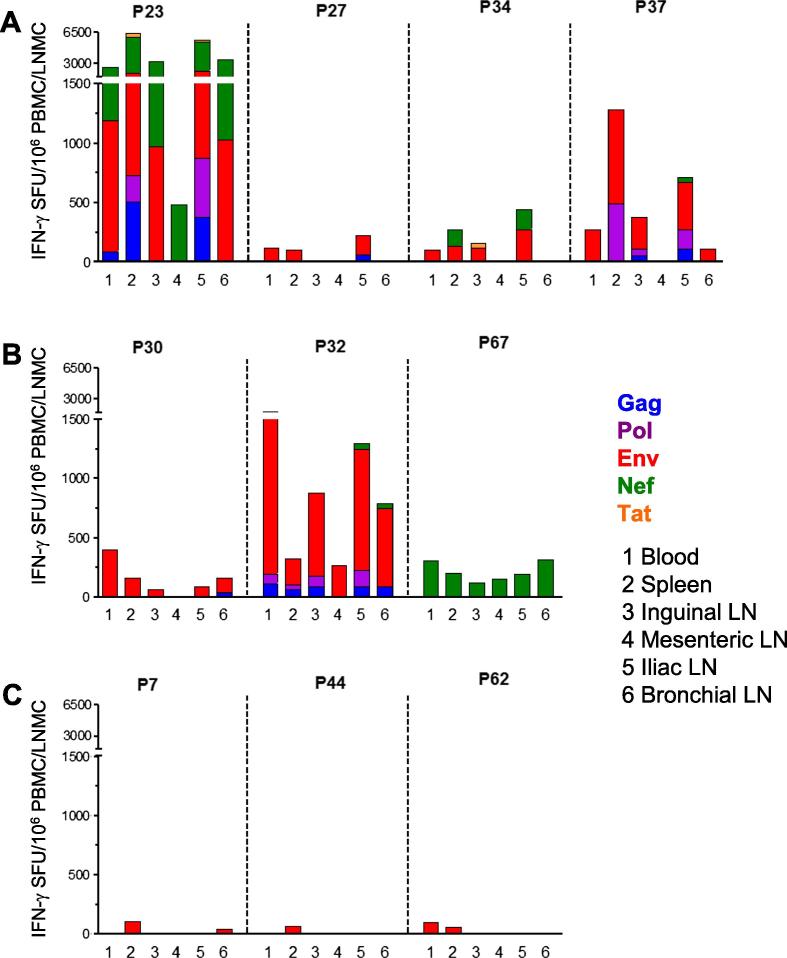
Comparison of systemic and mucosal lymph node T cell responses in rhesus macaques after vaccination with MVA-C and gp140Env/MF59. Blood, spleen and a variety of lymph nodes were harvested at euthanasia either 1 (T9) (A) or 12 (T11) (B) weeks after MVA-C/Env administration and the IFN-γ ELISPOT responses were measured using freshly isolated mononuclear cells. The same tissues were collected from a control group (C) at 1 (P62) or 12 (P7, P44) weeks after vaccination with gp140Env/MF59 alone and analyzed in an identical manner. The PBMC data in (A) are the same as that shown in Fig.1D (T9) as this was the time of euthanasia, whilst (B) shows T11 PBMC data, not depicted in Fig.1D. Cells (200,000 per well) were stimulated with HIV peptide pools as described, and cumulative responses are expressed as spot forming units (SFU) per million mononuclear cells. A cut-off value of 40 SFU for positive responses was used [background responses (unstimulated cells) were an average of 17 SFU/10^6^ cells, standard deviation: 7]. Medians are shown as a horizontal line. Statistical comparisons were determined using a non-parametric Mann-Whitney test.

To investigate T cell responses at mucosal sites, animals were euthanized at 1 or 12 weeks after MVA-C/Env vaccination, as indicated in Fig.1A, and several lymph nodes were analyzed. Animals in Group A showed HIV-specific cellular immune responses in multiple lymph nodes, including those draining the mucosa of the gut (mesenteric), genital tract (iliac and inguinal) and lungs (bronchial) (Fig.2A and B). Notably, cellular responses were detected at both 1 (Fig.2A) and 12 weeks (Fig.2B) after vaccination. Responses in iliac lymph nodes were detected in 7/7 MVA-C/Env protein vaccinated animals ranging from 84 to 5586 (median: 444) SFU/10^6^ cells, whereas 3/7 animals showed responses in mesenteric lymph nodes (median: 270; range: 152–482 SFU/10^6^ cells). Responses in bronchial lymph nodes were detected in 5/6 animals (median: 320; range: 113–3460 SFU/10^6^ cells) while 6/7 animals had responses in inguinal lymph nodes (median: 269; range: 62–3299 SFU/10^6^ cells). All animals (7/7) had responses in the spleen ranging from 105 to 6397 (median: 273) SFU/10^6^ cells. In general, responses in blood were of a similar specificity as those in spleen and lymph nodes draining mucosal compartments, with 3/7 animals displaying additional specificities in spleen (Fig.2A and B). In addition, the majority of animals had substantially different response magnitudes in spleen compared to blood, with 3/7 animals with greater than twofold higher responses, 2 animals with responses twofold lower than blood, and 2 animals with responses of similar magnitude in spleen and blood. These differences may reflect different numbers of CD4 or CD8 T cells in splenocytes compared to PBMC and the dynamics of the immune response. In 6/7 animals, Env-specific T cell responses were detected in multiple lymphoid tissues, including iliac lymph nodes. Unsurprisingly, the three animals in Group B vaccinated with the protein vaccine alone generated only modest Env-specific ELISPOT responses ranging from 57 to 108 SFU/10^6^ cells restricted to the spleen (3/3), blood (1/3) and bronchial lymph nodes (1/3) (Fig.2C). Thus, these data indicate that the vaccine regimen of poxvirus and protein, with prior DNA and poxvirus priming, generated T cell immunity in a broad range of lymphoid tissues, including high magnitude responses directed at multiple HIV antigens.

### 3.3 MVA-C vaccination and gp140Env/MF59 induce Env-specific binding antibodies

**Fig. 3:**
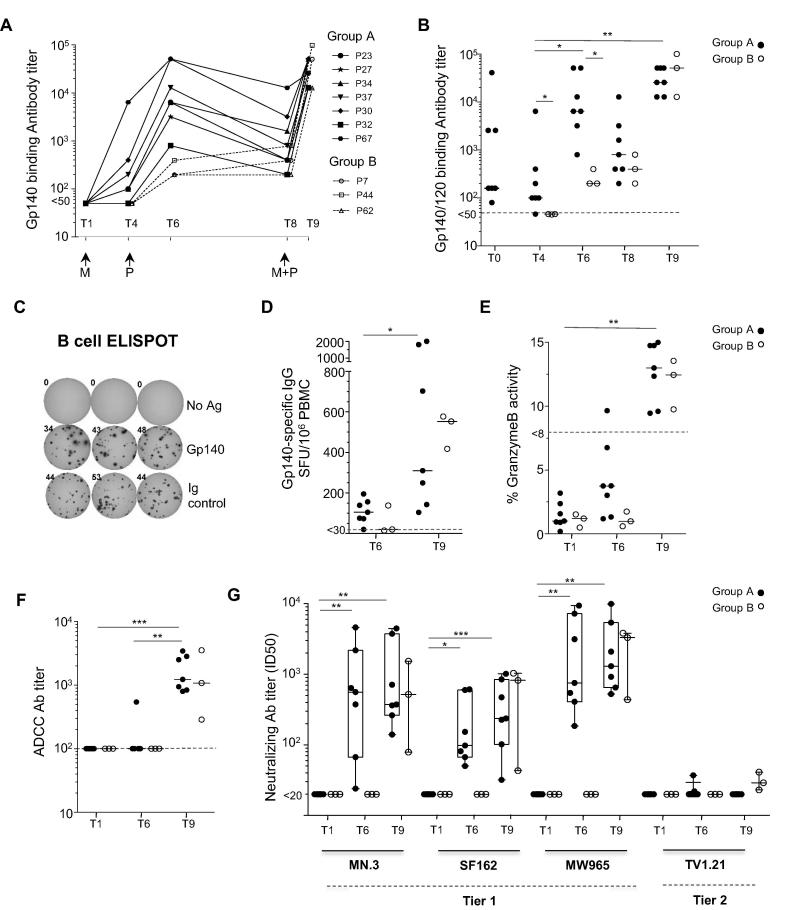
Humoral responses induced by MVA-C and gp140Env/MF59 vaccination in the blood of rhesus macaques. Endpoint titers and functional activity of antibody and B cell responses were measured in all animals. (A) Gp140-specific binding antibodies detected in sera obtained at baseline (T1) representing the time point when Group A animals received MVA-C re-vaccination, 4 weeks after MVA-C (T4) just before boosting with gp140Env/MF59 (both groups) and various time points as depicted in Fig.1A above. T8 time point represents the time for boosting with either gp140Env/MF59 (Group B) or combined MVA-C and gp140Env/MF59 (Group A) and T9 is 1 week after this vaccination. Values represent endpoint titers as measured in a standard ELISA assay with Gp140 (TV1) protein which was identical to the vaccine Gp140 protein. Antibody titers of <50 were considered negative. (B) Statistical comparisons of endpoint titers in both groups at T4, T6, T8 and T9. Antibody titers <50 (dotted line) were considered negative. T0 is included for comparison, representing Group A endpoint titers to Gp120 after DNA-MVA vaccination 3.5 years prior to re-vaccination. (C) A representative B cell ELISPOT assay showing wells that were coated with ‘No Antigen’ (No Ag; saline only as negative control), gp140Env protein (Gp140) or anti-human IgG monoclonal capture antibody (Ig control; as positive control) and incubated for 24 h with PBMC that had been stimulated for 5 days with a B-cell poly-activator R848 (TLR 7/8 agonist). (D) Frequency of Gp140-specific IgG-producing B cells per 10^6^ PBMC, measured at T6 and T9. A cut-off value of 30 SFU/10^6^ PBMC for positive responses is indicated with a dotted line. (E) ADCC activity of vaccine-induced antibodies. Granzyme B (GzB) activity reported as maximum percentage GzB activity detected using gp120-coated target cells, measured in serum from T1, T6 and T9. The results were considered positive if the percentage GzB activity after background subtraction was >8% (dotted line). (F) ADCC antibody titers measured in serum at T1, T6 and T9, detected in the ADCC-luciferase assay. Antibody titers of <100 (10^2^; dotted line) were considered negative. (G) Neutralizing antibody titers against Tier 1 (MN.3, SF162 and MW965) and Tier 2 (TV1.21 and DU151.2) viruses, determined using the TZM-bl assay. (Results with DU151.2 were negative and thus not shown). Lines indicate medians and box and whiskers indicate the interquartile range and range for positive responders. Statistical comparisons were performed using a non-parametric one-way ANOVA Friedman test with Dunn’s post test for multiple comparisons. *: p < 0.05, **: p < 0.01; ***: p < 0.005.

We next examined the ability of the vaccine regimen to induce HIV-specific antibody responses. As shown in Fig.3A, Env-specific binding antibodies were undetectable at the time of MVA-C re-vaccination (T1), but increased modestly in 6/7 animals after MVA-C vaccination (T4). There was a further and significant increase in median titer to >10^3^ after the first protein vaccination (Fig.3A and B; T6). In contrast, in the 3 animals in Group B that received protein only, Env-specific antibody titers were modest (<10^3^) and significantly lower than Group A after the first gp140Env/MF59 vaccination (Fig.3A and B). Peak titers in Group A were greater than those at T0 (measured to gp120), the time point after DNA-MVA vaccination 3.5 years earlier. A similar outcome was reflected in the B-ELISPOT assay (Fig.3C and D), which showed a modest magnitude of Env-specific IgG-secreting plasma cells at T6 for 6/7 animals in the MVA-C/protein group compared with only 1/3 animals in the protein-only group. The antibody titers increased substantially to >10^4^ after the final immunization of MVA plus protein for Group A, and protein-only for Group B (Fig.3A and B), and there was no difference in binding antibody titers between the two groups 1 week after the final immunization (T9). The B-ELISPOT assay showed an increase of >3-fold in median magnitudes of Env-specific IgG-secreting cells at T9 (Fig. 3B and D). Env-specific IgG-secreting plasma cells did not correlate with antibody binding titers (data not shown). Taken together, these results indicate that both a poxvirus-protein or protein-only regimen induce high titers of Env-specific binding antibodies.

### 3.4 MVA-C vaccination synergizes with gp140Env/MF59 for induction of Env-specific antibodies that mediate ADCC and Tier 1 neutralization

To determine whether functional antibodies were induced by the candidate vaccine regimens, the presence of Env-specific antibodies with the capacity to mediate ADCC or neutralize HIV was investigated. ADCC capacity was demonstrated in all animals in both groups after the final immunizations, as measured by Granzyme B activity (Fig.3E) [36], with an ADCC antibody titer of 10^3^ in both groups (Fig.3F) [21]. Thus, two protein immunizations were required for the development of ADCC, whether in combination with MVA-C or not.

Next, the presence of neutralizing antibodies (Nab) against Tier 1 and 2 HIV-1 viruses was investigated in a standard TZM-bl assay [35]. After the first gp140Env/MF59 vaccination at T6, Nab were detected in the sera of the MVA-C-vaccinated group, but not the protein-only group (Fig.3G). For MVA-C immunized animals, the single boost with gp140Env/MF59 protein vaccine induced higher titer Nab against multiple Tier 1 viruses (MN.3, SF162, MW965). Only 1/7 Group A animals displayed detectable and low titer neutralization against TV1.21, the vaccine strain, and only at T6 (Fig.3G). Following the final immunization, Nab titers against Tier 1 viruses were similar in both groups, and all 3 animals in the protein-only group developed low titer Nab to TV1.21. No detectable Nab activity against DU151.2, a Tier 2 clade C virus expressed by MVA-C, was observed in any animal (data not shown). These data demonstrate the ability of both vaccine regimens to induce functional antibodies capable of mediating ADCC and Tier 1 neutralization.

## 4 Discussion

We have previously reported on the immunogenicity of DNA-C and MVA-C and shown that a DNA prime-MVA boost immunization induces robust vaccine-specific cellular responses in the mouse and nonhuman primate models [26], [28], [32]. Here, we show that (i) a single vaccination with MVA-C elicits immune responses in rhesus macaques previously vaccinated in a prime-boost combination with a DNA-C and MVA-C vaccine regimen 3.5 years earlier; (ii) an envelope protein vaccine elicits functional antibody responses in peripheral blood, when added as a booster to a DNA prime-MVA boost or protein alone regimen; and (iii) concurrent administration of MVA-C and gp140 Env/MF59 induces cellular responses in multiple mucosal lymph nodes.

It may be anticipated that the first generation of successful candidate HIV vaccines will require booster vaccinations after the initial vaccine regimen to ensure continuous protection. The issue of whether a candidate HIV vaccine can efficiently prime long-term booster vaccinations has not been sufficiently addressed. In this NHP study, administration of a single MVA-C booster after >3.5 years increased the magnitude of cellular responses from below the level of detection to high magnitude responses one week after vaccination. These responses displayed a similar magnitude and breadth as the initial DNA/MVA vaccination, indicating that a population of long-lived memory T cells induced by the initial vaccine regimen, had persisted in these animals for at least 3.5 years. This finding supports the use of a poxvirus-vectored vaccine for long-term boosting, and suggests that further research to examine the longevity, phenotype and frequency of memory T cells that persist following a DNA/MVA vaccine regimen is required. Due to limited material, the phenotype of memory T cells present at the baseline was not examined. Both CD4+ T central and effector cells have been associated with virus control in HIV controllers [37] whilst memory CD8+ T cells have also been associated with low viral set points in early HIV-1 infection [38], indicating the importance of vaccine-generated memory T cells. Furthermore, vaccine-induced effector memory T cells have been shown to protect vaccinated NHP against mucosal SIV challenge [7], [8] and even to lead to immune clearance over time [7], [8].

The study of mucosal transmission of SIV in macaques has illustrated that after the infection of mucosal target cells, the virus rapidly disseminates to regional lymph nodes before reaching distal sites [39], [40], signifying the importance of inducing immunity at MALT. In this study, we demonstrate the presence of vaccine-induced cellular responses in various lymphoid tissues following systemic immunization with a MVA/protein vaccine regimen. Notably, for all animals immunized with an MVA/protein vaccine regimen, cellular responses were present in the iliac lymph node, which is a key node draining the genital tract. The data from our NHP study suggests potential induction of cell-mediated responses at mucosal lymphoid sites of vaccine recipients. These findings are relevant to the recently completed HVTN 073E/SAAVI 102E clinical trial in which the SAAVI vaccine regimen was combined with the gp140 Env/MF59 protein in a similar vaccination regime [41], but vaccine immune responses in the mucosal compartment were not analyzed in this phase 1 clinical trial. Protection against mucosal SIV challenges has been attributed to SIV-specific CD4+/IL2+ T cells in colorectal tissues and CD8+/IFN+ T cells in the vaginal tissue [42], signifying the potential role of vaccine-induced mucosal cellular responses for a HIV vaccine.

Some studies have demonstrated that ADCC activity, a non-neutralizing antibody function mediated largely by Env-binding antibodies [43] contributed to viral control and protection against SIV in macaques [17], [44] and reduced risk of HIV-1 acquisition in the RV144 study [13], [45]. ADCC responses are therefore considered to be a desirable response for candidate HIV vaccines [13], [46]. In the current study, we found ADCC activity in the sera of all animals after immunization with a second Env protein boost in both the MVA/protein and the protein-only groups, indicating the importance of a protein immunogen in the elicitation of an ADCC response. An early low ADCC response was observed in 1 of 7 MVA-C vaccinated animals but not in the protein-only group, suggesting that T cell priming had a positive impact on the induction of ADCC-mediating antibodies. This suggestion was also supported by the observation that the median binding antibody titer in the sera of MVA-C vaccinated animals was significantly higher than that of the protein-only group after the first protein boost, although the levels were similar in both groups after the final immunization. Binding antibodies directed at the V1/V2 region of gp120 were found to be a correlate of protection in RV144 trial [13]. Here, we used a V2-deleted gp140 and thus could not assess the ability of the vaccine regimen to induce these antibodies. Env proteins including V2, as well as more native stabilized trimeric Env proteins, could potentially induce these and broadly neutralizing antibodies to HIV [47].

Although induction of an effective non-neutralizing antibody response is thought to be beneficial in a HIV vaccine [10], [13], [45] only neutralizing antibodies have been demonstrated to directly block HIV transmission [48], [49]. In our NHP study, we observed a 100% (7/7) response rate for the MVA-C vaccinated group with respect to Nab against the 3 Tier 1 viruses (MN.3, SF162 & MW965) following the first protein boost (no detectable Nab were observed after the original DNA/MVA regimen; data not shown). A similar response was only seen in the protein-only group after the second protein boost. The HVTN 073E/SAAVI 102E study which evaluated a DNA/MVA plus Env protein boost in humans also observed a 56.2% (9/16) and a 100% (15/15) response rate against MW965 after the first and second protein boost respectively [41]. Both studies suggest that the priming with a DNA/poxvirus vaccine regimen induced T cell responses that influenced the ability to generate neutralizing antibodies, as seen in the RV144 study [12]. Circulating blood T follicular helper CD4+ cells have been linked to the ability to produce bNab in HIV-infected people [50] and SHIV-infected NHP [23]. Preliminary results of RV305 and RV306 which were follow-up studies of RV144 have recently been reported [51]. These follow-up studies investigated additional boosting with either a concurrent poxvirus/protein or protein alone given 48–72 weeks (RV306) or 6–8 years (RV305) after the first vaccination in RV144. In general, these preliminary studies shows boosting of binding IgG antibodies to gp120 and V1/V2 and Nab against Tier 1 viruses compared with RV144 results, with better boosting effects seen with delayed booster inoculation. Our NHP study suggests the possibility of long-term boosting of mucosal cell-mediated responses as well by concurrent poxvirus/protein vaccine strategies.

Our study had several limitations. Our control group of animals was given gp140 Env/MF59 protein, whereas an additional control group would have been one that had been given rMVA at the initial time point, and a combination of protein and rMVA at the later time point. In future work, we could perform flow cytometric analyses to determine whether responses were mediated by CD4 or CD8 T cells, and their polyfunctional nature. Furthermore, optimally defined prime-boost intervals could potentially improve vaccine responses obtained [52]. Lastly, Env and Nef cellular responses dominated the vaccine response, and further strategies to boost Gag responses, that have demonstrated an enhanced antiviral potency compared to other specificities, may be needed.

In conclusion, we show that after 3.5 years, a single MVA-C vaccination was able to rapidly elicit T cell responses in the blood and elicit similar responses in the mucosal-associated lymph nodes of animals previously vaccinated with DNA and MVA-based vaccines. The combination of DNA, MVA and Env protein vaccine candidates based on HIV-1 subtype C was highly immunogenic in rhesus macaques, with a gp140 Env protein boost capable of inducing functional antibodies to HIV. This study showed that, whilst induction of antibodies capable of mediating ADCC and virus neutralization needed a protein immunization, T cell priming vaccines are required to induce cellular responses in mucosal-associated lymph nodes. Although early humoral responses to Env differed between and MVA/protein and the protein-only group, responses were similar in both groups after the second protein booster, suggesting that MVA vaccination did not provide a benefit to functional humoral immunity. However, these antibody responses may be augmented by the higher magnitude and broad T cell responses in the MVA/protein group and enhance protective immunity to HIV. This study further justifies the use of poxviruses as vaccine vectors in combination with HIV envelope-based immunogens in developing future candidate HIV vaccine regimens that are targeted at conferring effective protection in the blood and mucosal sites of viral entry.

## Declaration

SWB is a shareholder of GSK. This does not alter her adherence to this journal’s policies on sharing data and materials. All other authors have no conflict of interest related to this work.

## Contributions

Conceived and designed the experiments: A-L.W., C.W., C.M.G., E.G.S. Performed the experiments: G.K.C., W.A.B., T.L.M., G.F., D.M. Analyzed the data: G.K.C., W.A.B., D.M., G.F. Contributed reagents/materials/analysis tools: S.W.B. Wrote the paper: G.K.C., W.A.B., A-L.W. All authors reviewed and approved the final manuscript.
